# Examining the online reading behavior and performance of fifth-graders: evidence from eye-movement data

**DOI:** 10.3389/fpsyg.2015.00665

**Published:** 2015-05-28

**Authors:** Yao-Ting Sung, Ming-Da Wu, Chun-Kuang Chen, Kuo-En Chang

**Affiliations:** ^1^Department of Educational Psychology and Counseling, National Taiwan Normal UniversityTaipei, Taiwan; ^2^Graduate Institute of Information and Computer Education, National Taiwan Normal UniversityTaipei, Taiwan

**Keywords:** comprehension process, online reading, hypertext, reading strategy, eye movement

## Abstract

Online reading is developing at an increasingly rapid rate, but the debate concerning whether learning is more effective when using hypertexts than when using traditional linear texts is still persistent. In addition, several researchers stated that online reading comprehension always starts with a question, but little empirical evidence has been gathered to investigate this claim. This study used eye-tracking technology and retrospective think aloud technique to examine online reading behaviors of fifth-graders (*N* = 50). The participants were asked to read four texts on the website. The present study employed a three-way mixed design: 2 (reading ability: high vs. low) × 2 (reading goals: with vs. without) × 2 (text types: hypertext vs. linear text). The dependent variables were eye-movement indices and the frequencies of using online reading strategy. The results show that fifth-graders, irrespective of their reading ability, found it difficult to navigate the non-linear structure of hypertexts when searching for and integrating information. When they read with goals, they adjusted their reading speed and the focus of their attention. Their offline reading ability also influenced their online reading performance. These results suggest that online reading skills and strategies have to be taught in order to enhance the online reading abilities of elementary-school students.

## Introduction

The development of technology and the growing popularity of the Internet have resulted in learners increasingly acquiring new knowledge and skills via the Internet. Offline reading tends to consist of left-to-right and top-to-bottom scanning in Taiwan (the official language is Traditional Chinese). By contrast, when a text is read online, reading paths may no longer be linear, instead involving jumping between different parts of the text. As they read, readers can click on the various links and decide for themselves what information to obtain. This new style of reading is developing at an increasingly rapid rate.

The phrase “offline reading” refers to the reading of printed, non-digital content in the form of traditional media such as books, newspapers, and magazines. With the development and popularization of computer technology and the Internet, information can now be transmitted using non-traditional media; one of the new such ways of transmission is online reading, whereby readers read information on the World Wide Web in various formats, including hyperlinks, texts, pictures, animations, sounds, and video. Readers have to read and understand the information in various forms and construct the meanings of webpage content (Rasmusson and Eklund, 2013). The Internet compresses time and space and enables people to share information and interact with each other in real time, which makes it a particularly useful learning tool (Coiro, 2007). Knowledge can be used, transmitted, and shared through the Internet, and so online reading is not merely a gateway to diverse resources, but is also characterized as real-time, interactive, open, and borderless.

Unlike traditional offline reading, online reading provides a “hypertextual” form of reading. The hypertext on the Internet is a type of text that uses a system of nodes and hyperlinks. The *node* is the basic unit of a hypertext system and often represents a concept or an idea. The nodes are connected by hyperlinks, and every node can be connected to an endless number of other nodes, thus forming a complex web-like structure. Information in a hypertext is usually presented in a non-linear fashion and does not follow a specific order: readers can connect to different nodes as they wish, and browse, search, and read them in any order. The same hypertext may therefore be read in different orders by different readers, and the same readers may read in different orders at different times. Unlike hypertexts, linear texts have nodes that connect with each other in a specific order: every node connects up or down to another node, and the information is structured in linear fashion, as in a book. When reading a linear text, readers can only read according to the order of the nodes.

The Internet provides access to a vast amount of information, and so finding relevant information in an effective way requires new online reading comprehension skills and strategies (Henry, 2006). Coiro and Dobler (2007) pointed out that, while reading comprehension processes for online and offline reading are similar in many respects, there are also some important differences. For example, hypertexts contain many hyperlinks, and readers have to play a more active role in deciding what to read next, instead of reading in the order dictated by the author. In addition, hypertexts lack clear textual context (e.g., a table of contents) as provided in printed books, so readers have to determine the relationships between the links for themselves (Yang, 1997; Balcytiene, 1999). The hyperlinks in hypertexts can appear in both text and images, and readers also have to interpret and integrate these visual cues (Kinzer and Leander, 2003). The intertexual connections in hypertexts are easily recognizable and accessible, and these further increase the complexity of the texts for readers.

Generally speaking, online reading comprehension involves five major functions: identifying important questions, locating information, analyzing information, synthesizing information, and communicating information (Leu et al., 2007, 2008). Online reading comprehension starts with the identification of an important question. This information is then searched for by entering keywords in a search engine. When the search results appear, webpages are selected that are likely to contain the desired information according to certain clues. Hyperlinks are clicked to open the webpages, and the relevance of their contents to the initial question is assessed and the accuracy and reliability of the information are verified. The reader normally needs to integrate the information in multiple discontinuous webpage texts in order to obtain an answer to the original question. Lastly, the information is communicated, discussed, and shared with other people.

Readers may encounter certain difficulties in online reading; for example, their search processes may be inefficient (Bilal, 2000; Eagleton, 2003). Other problems include that the reader's search question may change during the browsing process (Lyons et al., 1997), or that when readers actually acquire the information they want, they might not know how to use it (Wallace et al., 2000). The non-linear reading of hypertexts can generate two problems: disorientation and cognitive overload (Conklin, 1987). Disorientation is due to the inherent nature of hypertexts making it possible for readers to become lost in the text and fail to obtain an overview of the whole—they do not know where they are within the network. This may also drive readers to roam around the information without knowing what to do next; readers therefore need high meta-cognitive abilities, and those with less adequate linguistic skills may become confused more easily. Cognitive overload refers to the hyperlink decisions that readers have to make when they browse texts by jumping from one link to another or when they engage in multilayer reading. This requires additional thought and attention to decide which browsing path to take, whether to follow up on a subtopic or to return to the previous topic, and how to deal with complex information choices. Excessive information may also cause readers to forget what they have read.

A good reader adopts different reading strategies in response to different reading goals and reading materials in order to optimally grasp the text's meaning and extract its information (Pressley and McCormick, 1995). Afflerbach and Cho (2009) considered that the greatest difference between offline and online reading is that the latter involves the complex Internet hyperspace in which readers must determine what texts are available and in what order they should be consulted. Coiro and Dobler (2007) reported that skilled sixth-grade readers reading Internet texts have to deal with more complex processes and choices than if they obtained the same information offline. Although hypertexts are defined as non-linear and hence do not follow a specific order, many readers still read them in a more or less linear fashion, merely directly transferring the skills and strategies used in offline reading to online reading, instead of using specific strategies that are more effective for online reading. Students need to be taught to use appropriate strategies and navigation skills in order to achieve their reading goals (Wu, 2014).

Schmar-Dobler (2003) compared offline and online reading using the following seven comprehension strategies developed by Pearson et al. (1992): ask questions, activate prior knowledge, monitor and repair comprehension, determine important ideas, synthesize, draw inferences, and navigate. Schmar-Dobler (2003) believed that the strategies of using prior knowledge, determining important ideas, synthesizing, and drawing inferences are similar to those used for offline reading. However, online readers must first understand these questions in order to avoid becoming lost or sidetracked. Online readers must perform a considerable amount of skimming and scanning to enable them to monitor and repair comprehension; such readers also often pursue navigation strategies that make use of features specific to the Internet, such as pop-up ads and link-based downloading, when searching for information.

Afflerbach and Cho (2009) divided online text comprehension strategies into eight phases. The first phases involve searching for and then selecting relevant websites and information retrieval systems in order to access and browse information related to the reading goals. The next phases involve generating keywords that will optimally narrow down possible information ranges, and examining hyperlinks based on the reading goals—before they begin reading, readers must first determine the usefulness and significance of the links (Kuiper et al., 2005). The next phases involve selecting and browsing hyperlinks related to the reading goals, and setting up a dynamic reading plan to achieve these goals. When faced with more than one hyperlink, readers can make inferences and predictions about the relative utility of the links, which requires rapid judgment based on minimal information (Leu et al., 2007); and before they start to read, they can make inferences about the relevance of the links on each page (Lawless et al., 2003). The reader can establish the reading order based on the coherence of the links and the relevance of the websites or webpages (Protopsaltis, 2008), and use modified or revised keywords to conduct further searches in order to acquire more appropriate links and reading paths (Salmerón et al., 2005).

Successful readers combine multiple appropriate keywords to narrow down the search range (Guinee et al., 2003). They also make use of information such as titles, subtitles, and addresses to assess and plan their browsing and reading paths among hyperlinks and websites, and to evaluate the relevance and quality of information in relation to their reading goals (Hill and Hannafin, 1997; Zhang and Duke, 2008). Such readers select appropriate links strategically and in a methodical way in order to proceed to the next stage of reading (Salmerón et al., 2005), and use prior knowledge such as specific reading paths based on prior experience of browsing certain websites (Lawless et al., 2003). Successful readers also plan, predict, monitor, and assess all of the strategies that they use (Coiro and Dobler, 2007).

On the other hand, inexperienced hypertext readers and Internet search novices are not familiar with hypermedia or the functions and rules of search engines (Eagleton, 2003; Kumbruck, 1998), which makes it difficult for them to acquire the information they seek on the Internet (Yang, 1997; Bilal, 2000; Henry, 2006). Unskilled readers cannot effectively narrow down the scope of their search questions, are likely to make hasty decisions that lead to them overlooking useful information, and do not know how to integrate different sources of information (Eagleton et al., 2003; Coiro and Dobler, 2004).

Coiro (2007) interviewed seventh-grade students with various degrees of competence in online reading. Interviewees were asked to think aloud so that the researcher could record features of their online reading comprehension strategies. When highly skilled readers searched for information, they tended to enter relevant keywords in appropriate places and were able to use several keywords in a flexible manner based on the task clues. By contrast, low-skilled readers tended to use “.com strategies” to try to guess website addresses or use irrelevant information in the task as clues and repeatedly use a single, ineffective strategy. Low-skilled readers were more likely to be sidetracked by information that they were interested in but which was irrelevant to the task. Highly skilled readers (1) adjusted their reading speed and read closely the information that was relevant to the task while skimming irrelevant information, (2) inferred the relevance of the information to the task from clues they found in webpages and used this to establish their online reading paths, (3) verified the accuracy of information by finding secondary online sources, and (4) thought about whether information was distorted because of an author's commercial interests. By contrast, low-skilled readers were not able to evaluate the accuracy of information by activating prior knowledge and they were also unaware of the need to verify this information. Clearly, there are significant differences in the online reading strategies used by students who have different online reading abilities.

Hypertexts represent an alternative educational learning medium to traditional paper-based texts. Although comprehending hypertexts requires the same cognitive processes involved in reading linear texts, hypertexts still demand additional cognitive processes (Salmerón et al., 2006). It has been proposed that learning is more effective when using hypertexts than when using linear texts (McDonald et al., 1990; Jonassen, 1993). However, other authors dispute this claim, arguing that readers are more likely to encounter difficulties when organizing the different parts of hypertexts (Salmerón et al., 2009), whereas all readers have to do to grasp the overall structure of a linear text is to follow the order of reading as laid down by the author (Britton, 1994). By contrast, with a hypertext the reader must make use of other textual features, such as graphical overviews or prior knowledge, in order to form a coherent representation of the text (Baccino et al., 2008). The first goal of the present study was to revisit this long-standing dispute by exploring the reading processes and learning performance of elementary-school students reading linear texts and hypertexts.

Previous studies of traditional reading forms have found that although readers with different abilities use the same strategies, highly skilled readers are more likely to use them to greater effect (Millis and King, 2001). Highly skilled readers tend to use diverse strategies to understand texts, while low-skilled readers rarely use reading comprehension strategies during the reading process (Stanovich, 2000). Highly skilled readers are better at integrating prior knowledge with the information in the text in order to improve their comprehension (Haenggi and Perfetti, 1992), whereas low-skilled readers lack relevant background knowledge and vocabulary and do not know how to use strategies correctly or how to choose and employ appropriate strategies in an efficient way (León and Carretero, 1995). However, few studies have compared the online reading processes and strategies of students with different offline reading abilities. Therefore, the second goal of the present study was to understand whether traditional offline reading abilities can also affect online reading processes and learning performances.

The most important difference between online and offline reading is that online reading comprehension always starts with a question (Leu et al., 2007). Taboada and Guthrie (2006) believed that the reading process differs depending on whether or not the posing of questions is involved. Online readers first have to understand the question in order to avoid becoming lost or sidetracked (Schmar-Dobler, 2003), they must construct keywords relevant to their reading goals in order to narrow down the vast possible scopes of information (Afflerbach and Cho, 2009), and having different online reading goals will also push them to adopt different reading strategies (Zhang and Duke, 2008). However, no previous study has directly investigated how the online reading process is influenced by reading goals. The third goal of the present study was therefore to compare the online reading processes of elementary-school students and their use of strategies in the presence and absence of specific reading goals.

To sum up, the present study sought to answer the following questions:

1. When students with high and low reading abilities read linear texts and hypertexts, do their reading processes differ depending on whether or not they have specific reading goals in mind when searching for and integrating information?
2. When students with high and low reading abilities read linear texts and hypertexts, do their reading performances differ depending on whether or not they search for and integrate information with specific reading goals in mind?

This study addressed these research questions by using eye-tracking technology and retrospective think aloud (RTA) techniques to examine the online reading behaviors of fifth-grade elementary-school students (henceforth referred to simply as “fifth-graders”). The study involved three variables: reading ability, reading goals, and text types. Studies of traditional linear texts have tended to use paper-based tests when assessing learner reading performance, but that methodology does not involve direct observation of the reading comprehension processes used by learners (Kaakinen et al., 2003). Hypertexts are read in a non-linear fashion, which makes it even more difficult to observe online reading processes using paper-based tests or by simply counting the number of clicks made on webpages and browsed items. By contrast, eye tracking allows inference of the relationship between reading and other cognitive processes by looking at readers' eye movements, such as fixation duration and fixation location (Just and Carpenter, 1980, 1987; Rayner and Pollatsek, 1987; Rayner, 1998). Hyönä and Lorch (2004) provided several reasons why eye tracking is a suitable tool for studying text processing: First, it can be used to explore online processing and provide indicators with high temporal and spatial resolutions, thereby revealing how readers process their reading across sentences (e.g., looking back at sentences they have already read). In addition, eye tracking does not disrupt the normal reading process and so allows for a near-natural learning environment in which learners are not disturbed and can read texts in whatever order they choose. The technology also records precisely in real time the eye movements and mousing behaviors of learners, making it a suitable tool for the study of online reading. RTA is a method of collecting spoken data from participants after their work is complete (van den Haak et al., 2003). This way while participants are working silently on their task they will not be negatively influenced, such as by being distracted. RTA works well with the eye tracker used in this study, because the experimenter can replay a participant's eye-tracking video and ask them what their thoughts were while performing the task.

## Methods

### Participants

The participants in this study were 50 fifth-graders from two elementary schools in northern Taiwan. The elementary-school Reading Comprehension Screening Test (RCST; Ko and Chan, 2006) was used as a screening tool (see below). The norm mean on fifth-grade examination paper B (see below) (*M* = 15.69) was used as a cutoff, with students scoring higher than the mean being classified as readers with a high reading ability (26 students, *M* = 20.08), and those scoring lower than the mean categorized as readers with a low reading ability (24 students, *M* = 12.25).

When the 50 participants had completed the online reading experiments, those whose eye-tracking sampling rate was lower than 35% (4 students) were excluded. Thus, 46 samples (23 boys and 23 girls, mean age 11 years) were analyzed, comprising 23 highly skilled and 23 low-skilled readers. On the other hand, online reading requires basic computer and internet skills. To control for this competency, the present study used a self-made information literacy test (ILT) to assess information literacy among the students (see below). It was found that the information literary did not differ significantly between the highly skilled readers (*M* = 12.57) and the low-skilled readers [*M* = 11.30; *t*_(44)_ = 1.25, *p* = 0.220].

### Research design

The present study employed a three-way mixed design: 2 (reading ability: high vs. low) × 2 (reading goals: with vs. without) × 2 (text types: hypertext vs. linear text). Reading ability was a between-subjects variable, with the elementary-school RCST used to divide students into groups with high and low reading abilities. The reading goals, divided into with and without goals, was a within-subjects variable. For the condition of reading with goals, students were given a set of questions in advance and then asked to find answers in the text, whereas no online-reading problem-solving task was given for the condition of reading without goals. The text types, divided into linear texts and hypertexts, was also a within-subjects variable, with each of the students given both linear texts and hypertexts to read.

The dependent variables were eye-movement indices and the frequencies of using online reading strategy. We used an eye tracker to record the eye movements of students as they engaged in online reading. The data gathered were then used to calculate three eye-movement indices: mean fixation duration (MFD), percentage of total viewing time (PTVT), and regression count (RC). The frequencies of using online reading strategy were calculated using an online-reading-strategy coding table compiled by the researcher (see Supplementary Material). We then analyzed the number of reading strategies that students used during the online reading process, including searching, free browsing, and comprehension monitoring. This study analyzed eye-movement tracking indices and the frequencies of using online reading strategy to assess the online reading processes used by the students. The students' online-reading problem-solving task scores were also analyzed in order to understand how they performed when they had specific reading goals.

### Measures

#### Elementary-school reading comprehension screening test

This study used a revised version of the RCST (Ko and Chan, 2006) as a screening tool to divide students into two groups: high and low reading abilities. The RCST is used to assess the reading comprehension abilities of elementary-school children, from second-graders to sixth-graders. The RCST consists of two parallel forms, A and B, for each grade. All examination papers consist of four types of questions (except for second-grade examination papers, which lack the first type): multi-meaning words, proposition integration, sentence comprehension, and short-text comprehension. The paper-based examination has four-choice questions. The present study used fifth-grade examination paper B, which contains 31 multiple-choice questions. Its internal consistency reliability coefficient (Cronbach's *α*) was.81, and the test-retest reliability coefficient was.85. In terms of validity, the test shows significant correlations with other reading comprehension tests and has a high criterion-related validity.

#### Information literacy test

The present study used the ILT specially designed for this study. The ILT was compiled on the basis of the following five core abilities of information education, as indicated in the curriculum guidelines for middle and elementary schools in Taiwan: understanding of the concept of information technology, use of information technology, information processing and analysis, understanding and using the Internet, and information technology and human society. The ILT consisted of 30 four-choice questions. Two language education experts and one elementary-school information technology teacher checked the test and provided suggestions on how to improve the clarity of expression of the items and the difficulty of information terminology. The test was carried out on 105 students, and its discriminability and reliability were then analyzed. The final version of the ILT comprised 20 questions with an internal consistency reliability coefficient of.81.

### Materials

#### Online reading materials

The present study selected natural-science articles that would be unfamiliar to elementary-school students. The articles came from the Internet, popular science books, and textbooks, and covered four areas: the formation and structure of the Earth, the composition of biological organisms, nanoscience, and constellations. Once the materials had been compiled, the researcher discussed them with several elementary-school teachers and invited two psychology experts to review them. Taking into account these expert opinions, the materials were revised and compiled into formal experimental materials comprising between 7900 and 8900 characters each.

**Figure 1:**
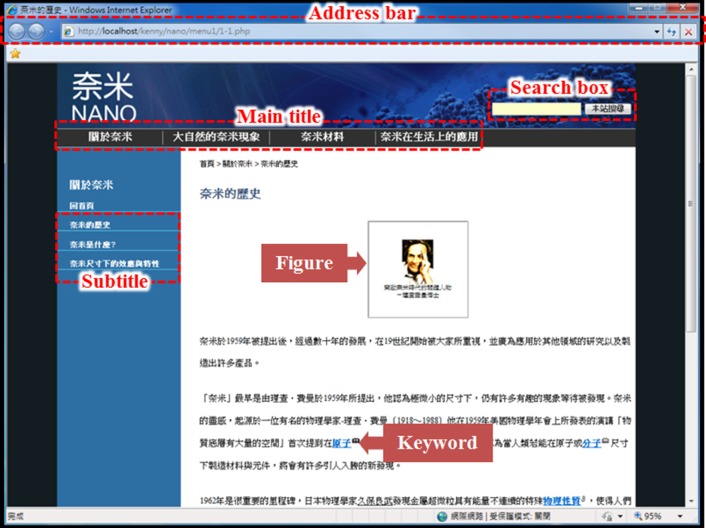
**Layout of our hypertext website**. This website contains different functionalities, such as a search box. Within the figure, we have highlighted all of the various areas of interest used in our data analysis, such as figures, titles, and keywords. This layout of our website also contains a variety of hyperlinks leading to various different topics.

**Figure 2:**
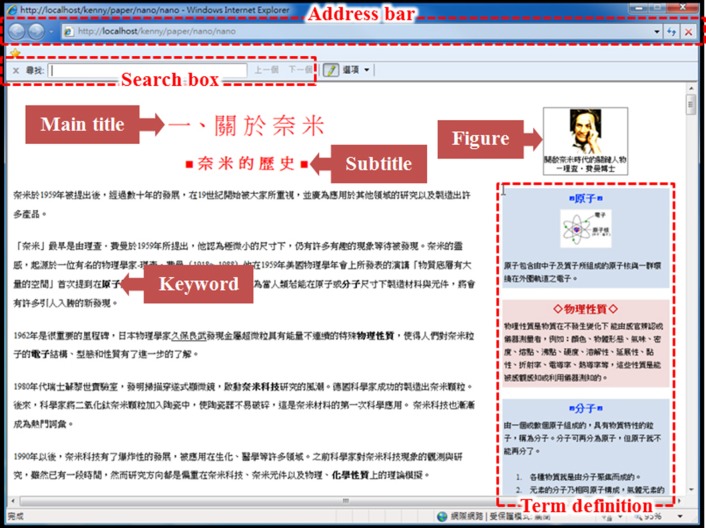
**Layout of our linear-text website**. This website contains different functionalities, such as a search box. Within the figure, we have highlighted all of the various areas of interest used in our data analysis, such as figures, titles, and keywords. This layout of our website contains no hyperlinks: All information related to the topic is included on this single page, accessible via a scroll-bar.

Both hypertext and linear-text Web versions of the articles were constructed (Figures 1, 2), and were viewed using the Internet Explorer 8 browser, with the tool bar retaining the “Previous Page,” “Next Page,” and “Refresh” buttons, and the address bar. The design of the hypertext and linear-text websites can be divided into three components: interface layout, search box, and hyperlinks. In the interface layout, the hypertext's main titles were located at the top of the webpage, reading from left to right, with subtitles listed on the left. When a main title was selected, the corresponding subtitles appeared. Article contents, including both texts and figures, were shown in the content area on the right-hand side of the webpage. In a linear text, the contents were listed in a single page according to the order of the main titles. A hypertext search box was located in the upper right-hand corner of the webpage, while in a linear text it was in the upper left-hand corner. Hypertext search results appeared as hyperlinks in the content area on the right, with partial webpage contents shown below the hyperlinks. In a linear text, the search results appeared as keywords in the text highlighted in yellow.

There were three types of textual hyperlinks—titles, term definitions, and definitions of difficult terms—and they were only available in the hypertexts (i.e., not the linear texts). The titles comprised the main titles and subtitles in the texts. Hypertext titles were blue and underlined, whereas in the linear texts they were presented in black standard font. Term definitions were definitions of natural-science terms displayed in blue with underlining, adjacent to small book icons. In linear texts, the term definitions were shown in boldface. Definitions were provided for difficult, more obscure terms that were unrelated to natural science, and were displayed in a blue font with underlining, adjacent to the clip icons. In linear texts, the definitions of difficult terms were shown in boldface. Detailed term definitions and definitions of difficult terms for the linear text appeared on the right-hand side.

#### Online-reading problem-solving task

To guide students to conduct goal-driven online reading, the present study designed an online-reading problem-solving task where students had to search for answers to specific questions. Each theme had its own task, and each student was randomly assigned two themes with tasks (goal-oriented reading) and two themes without tasks (reading with no specific goals) to allow comparisons (see Section Procedure for details). The task was designed so that it would give insight into the performances of students with different reading abilities when they search for and integrate information in different textual contexts. Two types of questions were asked about each topic: two multiple-choice questions and an essay question divided into two subquestions (see Supplementary Material). An example of the former is as follows:

“The physical properties of materials maybe change when they are reduced to the nanoscale dimensions. Which one of the following physical properties of materials *does not* change at nanoscale dimensions?”

An example of the essay questions is as follows:

“Give two examples of nanotechnology in nature and explain how they work. Also give two examples of everyday appliances that make use of nanotechnology and explain how they work.”

The students had to choose the best of the four answers in the multiple-choice questions. These questions were used to test whether students could find the information they needed by reading. The purpose of the essay question was to make it necessary for the students to integrate relevant information from different passages or webpages when constructing an answer. Multiple-choice questions are dichotomous response items while essay questions are polychotomous response items. Each essay script was scored by two markers; if their scores differed significantly, the script was assessed by a third marker. Each text contained two multiple-choice questions, and hence the accuracy score of these questions was (number of correct answers/2) × 100. The nanoscience essay question was scored out of 12 (two subquestions, each worth 6 points); the accuracy score for this question was therefore (score/12) × 100. The essay questions for the other three topics were each worth 8 points (two subquestions, each worth 4 points), so their accuracy score was (score/8) × 100. The overall accuracy score for each topic was determined as (accuracy rate of multiple-choice questions + accuracy rate of essay question)/2, which meant that the score for each problem-solving task ranged from 0 to 100.

### Apparatus

The non-intrusive Swedish-made Tobii X120 eye tracker was used. The camera and light source were set up under the screen on which images were displayed with a tracking distance of 50–80 cm between the screen and the subject's pupils. The Tobii X120 device allows for larger head movements and can track the subject's eyes for a long time without tiring them, and has a data sampling rate of 120 Hz. Tobii Studio software was used to control the experimental process and analyze the eye-movement data. In addition to collecting eye-movement data in real time, Tobii Studio can be used to record video, which is helpful for students to reflect on and describe their reading behaviors in subsequent interviews.

To reduce possible confounding factors, a simulated online reading website was used in this study. The website was hosted on a Fujitsu laptop computer with an Apache server and MySQL database. The website was presented on Acer 19-inch LCD monitors (resolution of 1280 × 1024 pixels and refresh rate of 60 Hz), and the participants viewed were provided with a keyboard and mouse.

### Data collection and analysis

#### Eye-movement data

The content that the students read can be divided into the following five areas of interest (AOIs):

1. Figures: pictures or tables used to explain or illustrate text content.
2. Titles: short phrases used to indicate text content and to organize and structure texts.
3. Topic sentences: sentences indicating the main concept of a passage, usually constituting the first one or two sentences in the passage.
4. Keywords: textual hyperlinks, term definitions, and definitions of difficult terms.
5. Paragraphs: parts of a text, and combinations of sentences that deal with the same topic.

The present study gathered data on three types of eye-movement indices in relation to the five AOIs: MFD, PTVT, and RC. The MFD is defined as the mean duration of all fixations within an AOI (Rayner, 1998). The total viewing time was computed by summing all fixation durations within an AOI; in order to exclude any effects of the total amount of reading, the total viewing time was expressed as a percentage rather than in seconds. Regression is defined as the reader's gaze returning to an area where it has already been (Rayner, 1998). The RC was defined as the number of times a reader returned to texts, figures, or hyperlink contents that had already been read, and may involve passages, chapters, or webpages. A 2 (reading ability) × 2 (reading goals) × 2 (text types) mixed ANOVA was applied to the three types of eye-movement indices.

#### Information on online reading strategies

This study investigated the frequencies with which students used three online reading strategies (searching, free browsing, and comprehension monitoring) based on the online reading comprehension process (Leu et al., 2007, 2008; Zawilinski et al., 2007) and offline and online reading strategies. In order to track the students' use of these strategies, we monitored their eye movements, mousing behaviors, and RTA techniques. For example, the search strategy was indicated by the keywords entered in the search box—these keywords might be copied from the text or formulated based on the text content. The free-browsing strategy includes skimming at random over unread texts or search results without reading sentences or paragraphs in their entirety.

Comprehension monitoring is how readers check to see if they actually understand the implications of the article. This includes goal setting, strategy selection, goal checking, and remediation (Gagne, 1985). These are all high-level cognitive functions, and are also known as metacognitive skills. Thus this study defines monitoring strategies as the reader's monitoring of article comprehension and the subsequent adjustment of reading behaviors, and includes the following four specific behaviors (which are also described in this study's online-reading-strategy coding table): (a) Assessing whether oneself understands the article; (b) figuring out the parts of an article one doesn't understand, such as by reading it again, slowing down one's reading speed, or finding/using other strategies; (c) asking a question of the article; and (d) identify confusing content and clarify doubts. This study, according to its RTA spoken data, observes whether the participants exhibited the above four behaviors to help understand the full extent of subject comprehension monitoring. A 2 (reading ability) × 2 (reading goals) × 2 (text types) mixed ANOVA was applied to the three online reading strategies.

### Procedure

This study was approved by the National Science Council, Taiwan. Consent was obtained from the parents of each participant before they participated in the experiment. Before the formal experiments, potential research participants were screened using the RCST and ILT. Pretests were run using the system software, webpages, and the experimental procedure to determine how the system software and webpages would run during the experiments. The duration that the participants spent on the whole practice, browsing, and answering the questions was also recorded for reference during the formal experiments.

Before beginning each formal experiment, the experimenter explained what the participant should pay attention to during the experiment. The seat was then adjusted for height and distance from the screen, such that the eyes were roughly 60 cm from the screen. The next step was calibration to ensure eye-movement data accuracy, after which the students were given one linear text and one hypertext, and 3 min to practice.

During the experiment, the participant was asked to read four texts in order. To avoid confounding effects of the order of presentation of textual themes, reading goals, and text types, the student was instructed to start with reading content that did not involve specific reading goals. The textual themes were arranged in the Latin square design. The participants were randomly assigned to one of eight groups (see Table 1): the order of text types was H-S-H-S for groups 1–4 and S-H-S-H for groups 5–8 (where H stands for hypertext and S for linear text).

**Table 1:** **Reading order in the formal experiments**.

**Group**	**Without reading goals**				**With reading goals**			
	**Text 1**		**Text 2**		**Text 3**		**Text 4**	
	**Theme**	**Type**	**Theme**	**Type**	**Theme**	**Type**	**Theme**	**Type**
1	A	H	B	S	C	H	D	S
2	D	H	A	S	B	H	C	S
3	C	H	D	S	A	H	B	S
4	B	H	C	S	D	H	A	S
5	A	S	B	H	C	S	D	H
6	D	S	A	H	B	S	C	H
7	C	S	D	H	A	S	B	H
8	B	S	C	H	D	S	A	H

*Themes: A, formation and structure of the Earth; B, composition of biological organisms; C, nanoscience; D, constellations. Types: H, hypertext; S, linear text*.

Participants performing the reading task without specific reading goals could decide for themselves when they had completed the reading, and no tests were administered thereafter. However, participants performing the reading task with reading goals had to first read the questions before they could start reading the texts to determine the answers. When they considered that they had found answers to the questions, the screen was turned off and they were required to answer the questions in the order, relying on their memories. The formal experiment ran for 70 min.

Once the formal experiment was completed, participants were shown the recording of their eye movements during the test and they were asked to perform an RTA exercise. They observed their behaviors and eye movements while the audiovisual record was played back and they retraced their thoughts during the task. The experimenter also posed questions to clarify any unclear or unusual points in order to obtain a better understanding of the thoughts and behaviors of the participants during the experiment.

## Results

### Analysis of eye-movement data

#### Mean fixation duration

Table 2 lists the MFDs for five AOIs: figures, titles, topic sentences, keywords, and paragraphs.

**Table 2:** **Mean and SD values of the MFD for each AOI (unit: milliseconds)**.

**AOI**	**Task type**	**High reading ability**				**Low reading ability**				***Post-hoc* comparison**
		**Linear text**		**Hypertext**		**Linear text**		**Hypertext**		
		***M***	***SD***	***M***	***SD***	***M***	***SD***	***M***	***SD***	
Figures	w/o goals	207	56	188	51	198	62	188	78	Interaction
	w/ goals	173	45	188	60	183	49	188	50	
Titles	w/o goals	192	61	168	114	178	46	179	63	Non-significant
	w/ goals	174	44	195	51	175	48	183	63	
Topic sentences	w/o goals	207	61	214	95	220	82	223	79	w/o goals > w/ goals
	w/ goals	186	48	186	51	190	56	186	45	
Keywords	w/o goals	212	61	228	119	212	104	231	128	w/o goals > w/ goals
	w/ goals	183	65	187	60	189	58	210	64	
Paragraphs	w/o goals	203	57	203	39	219	76	229	71	w/o goals > w/ goals
	w/ goals	181	47	183	45	181	53	188	42	

*w/o goals, without goals; w/ goals, with goals*.

For figures, the main effect of the reading-goals variable was statistically significant [*F*_(1, 44)_ = 4.79, *MSE* (mean squared error) = 1428, *p* < 0.05, η^2^ = 0.10]. The MFD was significantly higher when reading without goals (*M* = 195, *SD* = 63) than with goals (*M* = 183, *SD* = 52). There was a statistically significant interaction between the reading-goals and text-types variables [*F*_(1, 44)_ = 7.35, *MSE* = 940, *p* < 0.01, η^2^ = 0.14]. Further testing of the simple main effect showed that the MFD when reading without goals was significantly higher for linear texts (*M* = 203, *SD* = 59) than for hypertexts (*M* = 188, *SD* = 66). Moreover, when students read linear texts, the MFD was significantly higher when reading without goals (*M* = 203, *SD* = 59) than with goals (*M* = 178, *SD* = 48). However, there was no statistically significant main effect of other variables or interaction between variables (all *p* values > 0.30)^1^.

No statistically significant main effect or interaction was found for titles (all *p* values > 0.10).

For topic sentences, the main effect of the reading-goals variable was statistically significant [*F*_(1, 44)_ = 17.18, *MSE* = 2240, *p* < 0.001, η^2^ = 0.28]. The MFD was significantly higher when reading without goals (*M* = 216, *SD* = 81) than with goals (*M* = 187, *SD* = 50). However, there was no statistically significant main effect of other variables or interaction between variables (all *p* values > 0.50).

For keywords, the main effect of the reading-goals variable was statistically significant [*F*_(1, 44)_ = 7.20, *MSE* = 5193, *p* < 0.05, η^2^ = 0.14]. The MFD was significantly higher when reading without goals (*M* = 221, *SD* = 107) than with goals (*M* = 192, *SD* = 63). However, there was no statistically significant main effect of other variables or interaction between variables (all *p* values > 0.10).

For paragraphs, the main effect of the reading-goals variable was statistically significant [*F*_(1, 44)_ = 33.00, *MSE* = 1270, *p* < 0.001, η^2^ = 0.43]. The MFD was significantly higher when reading without goals (*M* = 213, *SD* = 63) than with goals (*M* = 183, *SD* = 47). However, there was no statistically significant main effect of other variables or interaction between variables (all *p* values > 0.20).

Reading speed patterns could be regarded as the extrinsic representation of reading actions (Liang and Huang, 2014). A 2 (reading ability) × 5 (AOIs) repeated-measures ANOVA was used to determine whether students with different reading abilities adjusted their reading speed according to the text content.

While analyzing the data for when students read linear texts without specific reading goals, we discovered the AOIs variable violated the assumption of sphericity (Mauchly's *W* =0.160, *p* <0.001). After Greenhouse-Geisser correction, the MFDs differed significantly between the five AOIs [*F*_(2.336, 102.778)_ = 5.81, *MSE* = 1940, *p* < 0.01, η^2^ = 0.12]. *Post-hoc* comparisons showed that the MFD was significantly higher for figures, topic sentences, keywords, and paragraphs than for titles, and significantly higher for topic sentences than for figures. However, there was no statistically significant main effect of the reading-abilities variable or interaction between variables (both *p* values > 0.10).

Similarly, we found in analyzing the data for when students read hypertexts without specific reading goals that the AOIs variable violated the assumption of sphericity (Mauchly's *W* =0.242, *p* <0.001). After Greenhouse-Geisser correction, the MFD also differed significantly among the five AOIs [*F*_(2.543, 111.905)_ = 4.94, *MSE* = 7984, *p* < 0.01, η^2^ = 0.10]. *Post-hoc* comparisons showed that the MFD was significantly higher for topic sentences, keywords, and paragraphs than for figures and titles. However, there was no statistically significant main effect of the reading-abilities variable or interaction between variables (both *p* values > 0.60).

We discovered the AOIs variable also violated the assumption of sphericity when analyzing the data for students who read linear texts with specific reading goals (Mauchly's *W* = 0.240, *p* < 0.001). After Huynh-Feldt correction, the MFD differed marginally significantly among the five AOIs [*F*_(2.688, 118.289)_ = 2.69, *MSE* = 849, *p* = 0.056, η^2^ = 0.06]. *Post-hoc* comparisons showed that the MFD was significantly higher for topic sentences than for figures, titles, and paragraphs. However, there was no statistically significant main effect of the reading-abilities variable or interaction between variables (both *p* values > 0.70).

No statistically significant main effect or interaction between variables was found when reading hypertexts with specific reading goals (all *p* values > 0.20).

#### Percentage of total viewing time

Table 3 lists the PTVTs for the five AOIs: figures, titles, topic sentences, keywords, and paragraphs.

**Table 3:** **Mean and SD values of the PTVT for each AOI (unit: %)**.

**AOI**	**Task type**	**High reading ability**				**Low reading ability**				***Post-hoc* comparison**
		**Linear text**		**Hypertext**		**Linear text**		**Hypertext**		
		***M***	***SD***	***M***	***SD***	***M***	***SD***	***M***	***SD***	
Figures	w/o goals	16.64	10.33	11.95	8.48	13.89	10.06	11.53	8.93	Non-significant
	w/ goals	14.93	10.33	12.95	6.87	16.18	11.16	15.29	9.23	
Titles	w/o goals	5.43	4.35	3.87	4.66	3.90	2.82	1.84	2.06	w/ goals > w/o goals
	w/ goals	6.05	3.64	8.16	7.11	6.98	6.22	5.79	4.71	
Topic sentences	w/o goals	20.42	5.95	13.79	9.00	23.03	6.61	15.38	9.65	Interaction
	w/ goals	27.50	11.34	21.25	7.69	24.73	7.74	15.99	7.93	Linear text > hypertext
Keywords	w/o goals	1.76	1.02	3.78	3.37	2.09	1.07	2.91	1.80	
	w/ goals	2.04	1.57	3.36	1.59	2.22	1.30	4.47	3.93	Hypertext > linear text
Paragraphs	w/o goals	55.75	11.14	66.60	14.62	57.08	9.84	68.33	14.28	w/o goals > w/ goals
	w/ goals	49.49	8.40	54.28	12.87	49.89	10.26	58.46	10.55	Hypertext > linear text

*w/o goals, without goals; w/ goals, with goals*.

No statistically significant main effect or interaction was found for figures (all *p* values > 0.10).

For titles, the main effect of the reading-goals variable was statistically significant [*F*_(1, 44)_ = 29.69, *MSE* = 13.79, *p* < 0.001, η^2^ = 0.40]. The PTVT was significantly higher when reading with goals (*M* = 6.75, *SD* = 5.66) than without goals (*M* = 3.76, *SD* = 3.85). However, there was no statistically significant main effect of other variables or interaction between variables (all *p* values > 0.10).

For topic sentences, the main effect of the reading-goals variable was statistically significant [*F*_(1, 44)_ = 11.05, *MSE* = 73.80, *p* < 0.01, η^2^ = 0.20]. The PTVT was significantly higher when reading with goals (*M* = 22.37, *SD* = 9.80) than without goals (*M* = 18.16, *SD* = 8.78). The main effect of the text-types variable was also statistically significant [*F*_(1, 44)_ = 45.61, *MSE* = 53.96, *p* < 0.001, η^2^ = 0.51]. The PTVT was significantly higher when reading linear texts (*M* = 23.92, *SD* = 8.57) than when reading hypertexts (*M* = 16.60, *SD* = 9.05). There was also a statistically significant interaction between the reading-ability and reading-goals variables [*F*_(1, 44)_ = 5.82, *MSE* = 73.80, *p* < 0.05, η^2^ = 0.12]. Further testing of the simple main effect showed that the PTVT when reading with goals was significantly higher for students with a high reading ability (*M* = 24.37, *SD* = 10.18) than a low reading ability (*M* = 20.36, *SD* = 8.97). Moreover, the PTVT was significantly higher when students with a high reading ability were reading with goals (*M* = 24.37, *SD* = 10.18) than without goals (*M* = 17.11, *SD* = 8.32). However, there was no statistically significant main effect of the reading-abilities variable or interaction between variables (all *p* values > 0.40).

For keywords, the main effect of the text-types variable was statistically significant [*F*_(1, 44)_ = 19.48, *MSE* = 6.07, *p* < 0.001, η^2^ = 0.31]. The PTVT was significantly higher when reading linear texts (*M* = 3.63, *SD* = 2.91) than when reading hypertexts (*M* = 2.03, *SD* = 1.27). However, there was no statistically significant main effect of other variables or interaction between variables (all *p* values > 0.08).

For paragraphs, the main effect of the reading-goals variable was statistically significant [*F*_(1, 44)_ = 29.51, *MSE* = 123.79, *p* < 0.001, η^2^ = 0.40]. The PTVT was significantly higher when reading without goals (*M* = 61.94, *SD* = 13.81) than with goals (*M* = 53.03, *SD* = 11.25). The main effect of the text-types variable was also statistically significant [*F*_(1, 44)_ = 30.14, *MSE* = 119.89, *p* < 0.001, η^2^ = 0.41]. The PTVT was significantly higher when reading hypertexts (*M* = 61.92, *SD* = 14.39) than when reading linear texts (*M* = 53.05, *SD* = 10.52). However, there was no statistically significant main effect of the reading-abilities variable or interaction between variables (all *p* values > 0.10).

#### Regression count

Table 4 lists the RCs.

**Table 4:** **Mean and SD values of the RCs of students with different reading abilities reading two types of texts with/without reading goals**.

	**Task type**	**High reading ability**				**Low reading ability**				***Post-hoc* comparison**
		**Linear text**		**Hypertext**		**Linear text**		**Hypertext**		
		***M***	***SD***	***M***	***SD***	***M***	***SD***	***M***	***SD***	
	w/o goals	0.44	0.97	0.96	1.52	0.39	0.82	0.91	1.53	Interaction
	w/ goals	4.96	4.52	8.22	5.42	4.74	6.19	10.74	7.10	

*w/o goals, without goals; w/ goals, with goals*.

The main effect of the reading-goals variable was statistically significant [*F*_(1, 44)_ = 89.36, *MSE* = 21.68, *p* < 0.001, η^2^ = 0.67]. The RC was significantly higher when reading with goals (*M* = 7.16, *SD* = 6.39) than without goals (*M* = 0.67, *SD* = 1.28). The main effect of the text-types variable was statistically significant [*F*_(1, 44)_ = 25.04, *MSE* = 12.19, *p* < 0.001, η^2^ = 0.36]. The RC was significantly higher when reading hypertexts (*M* = 5.21, *SD* = 6.34) than when reading linear texts (*M* = 2.63, *SD* = 4.47). There was also a statistically significant interaction between the reading-goals and text-types variables [*F*_(1, 44)_ = 17.34, *MSE* = 11.19, *p* < 0.001, η^2^ = 0.28]. Further testing of the simple main effect showed that the RC was significantly higher when reading with goals (linear texts: *M* = 4.85, *SD* = 5.42; hypertexts: *M* = 9.48, *SD* = 6.44) than without goals (linear texts: *M* = 0.41, *SD* = 0.90; hypertexts: *M* = 0.94, *SD* = 1.52), irrespective of whether linear texts or hypertexts were being read. At the same time, the RC when reading with goals was significantly higher for hypertexts (*M* = 9.48, *SD* = 6.44) than for linear texts (*M* = 4.85, *SD* = 5.42). However, there was no statistically significant main effect of the reading-abilities variable or interaction between variables (all *p* values > 0.10).

### Analysis of online reading strategies

Table 5 presents descriptive statistics indicating the frequencies of using the three strategies: searching, free browsing, and comprehension monitoring.

**Table 5:** **Mean and SD values of the frequencies of using three online reading strategies**.

**Strategy**	**Task type**	**High reading ability**				**Low reading ability**				***Post-hoc* comparison**
		**Linear texts**		**Hypertexts**		**Linear texts**		**Hypertexts**		
		***M***	***SD***	***M***	***SD***	***M***	***SD***	***M***	***SD***	
Searching	w/o goals	0.00	0.00	0.00	0.00	0.00	0.00	0.00	0.00	Non-significant
	w/ goals	0.00	0.00	0.26	1.22	0.00	0.00	0.22	1.02	
Free browsing	w/o goals	22.26	8.13	7.44	7.81	16.91	7.16	6.44	4.66	Interaction
	w/ goals	18.74	7.47	12.70	6.83	17.26	7.15	10.09	6.21	
Comprehension monitoring	w/o goals	0.22	0.51	0.04	0.20	0.04	0.20	0.04	0.20	
	w/ goals	0.22	0.51	0.13	0.45	0.26	0.67	0.00	0.00	Linear text > hypertext

*w/o goals, without goals; w/ goals, with goals*.

No statistically significant main effect or interaction was found for searching (all *p* values > 0.10).

For free browsing, the main effect of the reading-goals variable was marginally statistically significant [*F*_(1, 44)_ = 3.94, *MSE* = 79.37, *p* = 0.053, η^2^ = 0.08]. The number of times that a free-browsing strategy was used was significantly higher for students with a high reading ability (*M* = 15.28, *SD* = 9.46) than a low reading ability (*M* = 12.67, *SD* = 7.86). The main effect of the text-types variable was statistically significant [*F*_(1, 44)_ = 72.63, *MSE* = 58.74, *p* < 0.001, η^2^ = 0.62]. The number of times that a free-browsing strategy was used was significantly higher when reading linear texts (*M* = 18.79, *SD* = 7.78) than when reading hypertexts (*M* = 9.16, *SD* = 6.92). There was also a statistically significant interaction between the reading-goals and text-types variables [*F*_(1, 44)_ = 14.14, *MSE* = 29.71, *p* < 0.001, η^2^ = 0.24]. The simple main effect showed that the number of times that a free-browsing strategy was used was significantly higher when reading linear texts (without goals: *M* = 19.59, *SD* = 8.11; with goals: *M* = 18.00, *SD* = 7.35) than when reading hypertexts (without goals: *M* = 6.94, *SD* = 6.45; with goals: *M* = 11.39, *SD* = 6.65), irrespective of whether they read with or without specific goals. In addition, when students read hypertexts, the number of times they used a free-browsing strategy was significantly higher when reading with goals (*M* = 11.39, *SD* = 6.65) than without goals (*M* = 6.94, *SD* = 6.45. However, there was no statistically significant main effect of the reading-goals variable or interaction between variables (all *p* values > 0.09).

For comprehension monitoring, the main effect of the text-types variable was statistically significant [*F*_(1, 44)_ = 7.30, *MSE* = 0.11, *p* < 0.01, η^2^ = 0.14]. The number of times that students used a comprehension-monitoring strategy was significantly higher when reading linear texts (*M* = 0.19, *SD* = 0.51) than when reading hypertexts (*M* = 0.05, *SD* = 0.27). However, there was no statistically significant main effect of other variables or interaction between variables (all *p* values > 0.10).

### Analysis of online-reading problem-solving task

No online-reading problem-solving task was given to students reading without goals, so the present analysis relates only to scores for online-reading problem-solving tasks performed by students while reading with specific goals. Table 6 presents the descriptive statistics for this analysis.

**Table 6:** **Mean and SD values of online-reading problem-solving task scores for students with different reading abilities reading different types of texts**.

**Text type**	**High reading ability**		**Low reading ability**	
	***M***	***SD***	***M***	***SD***
Linear text	70.83	21.10	50.18	23.83
Hypertext	48.10	21.53	35.60	15.30

A 2 (reading ability) × 2 (text types) mixed ANOVA showed that the main effect of the reading-abilities variable was statistically significant [*F*_(1, 44)_ = 14.50, *MSE* = 552, *p* < 0.001, η^2^ = 0.25]. Task scores were significantly higher for students with a high reading ability (*M* = 60.51, *SD* = 24.76) than a low reading ability (*M* = 41.85, *SD* = 19.69). The main effect of the text-types variable was also statistically significant [*F*_(1, 44)_ = 18.50, *MSE* = 342, *p* < 0.001, η^2^ = 0.30]. Task scores were significantly higher for linear texts (*M* = 59.47, *SD* = 24.16) than for hypertexts (*M* = 42.89, *SD* = 21.31). There was no statistically significant interaction between reading ability and text types (*p* values > 0.20).

## Discussion and conclusion

This study used eye-tracking technology and RTA techniques to examine the online reading behaviors of fifth-graders. The main question that the study sought to answer was whether students with high and low reading abilities reading linear texts and hypertexts use different reading processes and exhibit different performances when searching for and integrating information with or without specific reading goals. The study sought to understand the online reading processes used by students by analyzing eye-tracking indices and the frequencies of using online reading strategy, and to assess their online reading performance by analyzing their online-reading problem-solving task scores.

### Online reading processes among fifth-graders

The MFDs for three AOIs—paragraphs, topic sentences, and keywords—were significantly higher when students read texts without specific reading goals than with goals. This suggests that students read more quickly to find answers when they read with goals for both linear texts and hypertexts. However, when reading figures the students adjusted their speed only when reading linear texts; that is, not when reading hypertexts. An increased reading speed might lead to students overlooking important information, which would increase their regression. Previous research showed that the students with higher reading speeds would reread the text more than once (Huang and Liang, 2014). The results show that the RC was significantly higher for students reading with goals than without goals, for both linear texts and hypertexts. It is possible that students still could not find the answers after performing rapid initial browsing, increasing the likelihood of them re-reading previous contents.

This study also found that, in the case of reading with goals, the RC was significantly higher when reading hypertexts than when reading linear texts. This reflects that disorientation or cognitive overload may occur during the non-linear reading of hypertexts (Conklin, 1987). When students are not entirely sure where they are within a hypertext network, they may roam about the hypertext in an unstructured way and repeatedly return to passages that they have already read. Previous studies have also found that students in the fifth and sixth grades are more likely to become lost in a non-linear and unfamiliar text structure (Chen, 2010). Moreover, when readers browse hypertexts by jumping between different passages, they are faced with the need to choose which hyperlinks to follow and which to ignore. Making such choices requires additional thinking and attention, and so fewer cognitive resources are available for other tasks. For example, the present study found that there was significantly less use of the comprehension-monitoring strategy when reading hypertexts than when reading linear texts, which shows that fifth-graders may not have sufficient cognitive resources to determine whether they understand the contents of hypertexts, and that they find ways to deal with content that they do not understand. By contrast, in order to grasp the overall structure of linear texts, readers only have to follow the order of reading as laid down by the author (Britton, 1994) and do not have to choose among multiple hyperlinks. Therefore, for students at this learning stage, searching for answers is easier in linear texts than in hypertexts.

Using search engines to narrow down the scope of reading should be an effective strategy to prevent disorientation or cognitive overload during information searches. However, almost none of the students included in the present study used this search strategy, even though the online-reading problem-solving task required them to search for answers. The survey revealed that 63.4% of the students knew that they could search using keywords, and while 54.3% of them saw the search engine, 44% of these students said they were not in the habit of using such a facility, and 36% said they did not know which keywords to use. This shows that students at this learning stage do not use the search strategy effectively (Chen, 2010). The desired information may be hidden deep in the text structure of hypertexts, and so would be inaccessible to the students unless they clicked on the appropriate hyperlinks. Previous studies have indicated that readers can use prior knowledge to guide their hypertext navigation (Lawless and Kulikowich, 1996; Barab et al., 1997) and find relevant resources (Yang, 1997; Balcytiene, 1999). But since the present study deliberately used reading materials that the included elementary-school students were unfamiliar with, it was difficult for them to use their prior knowledge to identify to relevant hyperlinks.

Coiro (2007) observed that highly skilled seventh-grade students adjusted their reading speed by closely reading information related to the task and skimming irrelevant information. Schmar-Dobler (2003) pointed out that readers adjust their reading speed according to their reading goals when reading offline, while for online reading they first have to perform a considerable amount of skimming. In light of this previous research, the present study also attempted to determine whether students with different reading abilities adjusted their reading speed according to the text content. It was found that when reading without goals, the MFDs were significantly higher for paragraphs, topic sentences, and keywords than for figures and titles, irrespective of the text type. This showed that students skimmed through figures and titles but slowed down when they read text content.

When the students read texts with specific reading goals in mind, they adjusted their reading speed in different ways, according to whether they were reading linear texts or hypertexts—with the former, the MFD was significantly higher for topic sentences than for figures and titles. It is also notable that the MFD was significantly higher for topic sentences than for paragraphs, which means that students were able to discriminate topic sentences from paragraph contents. Since topic sentences often indicate the main concept of a paragraph, this suggests that the students deliberately slowed down to read important information in topic sentences. By contrast, the MFD did not differ significantly between the five AOIs when reading hypertexts, which shows that when the students searched for answers in hypertexts they did not adjust their reading speed according to the text contents, and this may have had an indirect impact on their performance in the online-reading problem-solving task. This phenomenon also occurred in relation to the free-browsing strategy: students used the strategy more often when searching for answers in linear texts than in hypertexts; they quickly browsed through irrelevant information and used skimming more often.

In relation to the allocated reading time, it was also found that students were more likely to grasp topic sentences in linear texts. The data show that when the students with different reading abilities read topic sentences, the PTVT was significantly higher for linear texts than for hypertexts irrespective of whether or not the readers had specific goals. However, the PTVT was significantly higher for hypertexts than for linear texts when reading paragraphs, which shows that the students were more likely to grasp the main ideas of linear texts and more likely to focus on the secondary content of hypertexts.

The text type affected the length of time students spent reading topic sentences. We also found that the interaction between two other factors influenced the reading time of topic sentences. When reading without goals, students with high and low reading abilities spent almost the same amount of time reading topic sentences. However, when reading with goals—for example, when searching for answers to questions in the task—those with a high reading ability spent a significantly longer amount of time reading topic sentences, while there was no change in the amount of time among students with a low reading ability. This shows that students with a low reading ability did not make adjustments appropriate to their reading goals. This means that readers with a higher reading ability or better skills do tend to use such strategies (McNamara, 2007).

On the other hand, when students with different reading abilities read paragraphs, the PTVT was always significantly higher when reading without goals than with goals, irrespective of the text type. By contrast, when students read titles, the PTVT was significantly higher for reading with goals than without goals. Titles can be used to organize a text, and readers can use them to understand the overall text structure. Students reading with goals spent more time reading titles in order to see whether a given paragraph was relevant to the task questions and to decide whether to continue reading. This indicates that students adopted a more effective way to screen information when searching for target information.

When students read keywords, they spent significantly more time when reading hypertexts than when reading linear texts. Keywords in hypertexts appear as hyperlinks with adjacent icons and are in blue and underlined, whereas keywords in linear texts are not hyperlinks and appear in boldface. Compared to content words being presented in black standard font in linear texts, the keywords in hypertexts will be visually more distinctive and hence more likely to attract reader attention. Previous studies have shown that students at this learning stage are more likely to be influenced by cues such as lexical and typographical cues when selecting from webpage menus (Rouet et al., 2011).

### Online reading performance of fifth-graders

We analyzed the online-reading problem-solving task scores for students' online reading performances in order to understand how they searched for and integrated information when they had specific reading goals. Four aspects of the reading process were found to explain why students performed better when reading linear texts than when reading hypertexts:

1. They adjusted their reading speed to suit text contents only when reading linear texts; they also slowed down strategically to read the main ideas of texts.
2. They spent more time reading topic sentences when reading linear texts, which increased the likelihood of understanding the main ideas of linear texts.
3. Their regression was higher for hypertexts, which means that they were more likely to experience disorientation and cognitive overload when engaging in non-linear reading.
4. When reading linear texts, students used more free-browsing and comprehension-monitoring strategies in order to increase the efficiency of their search for information and to grasp the meaning of text contents.

Fifth-graders are more familiar with linear reading, and so they need to learn more skills for and acquire more experience of hypertext reading.

It was also found that the scores for the online-reading problem-solving task were significantly higher for students with a high reading ability than a low reading ability. According to eye-movement data, the PTVT of students with a high reading ability when reading topic sentences was significantly higher than that of students with a low reading ability, which shows that students with a high reading ability were more able to grasp the main ideas of texts. However, the MFD, RC, and frequency of use of online reading strategies did not differ significantly between students with high and low reading abilities. It is possible that the students with a high reading ability are better able to use higher level strategies and higher level comprehension processes, but no evidence of such differences was obtained in the present study. The RCST was used as a screening tool to test the offline reading comprehension of fifth-graders. This test consists of four types of questions: multi-meaning words, proposition integration, sentence comprehension, and short-text comprehension. Although the test is not designed as a diagnosis tool for online reading comprehension, many aspects of the reading comprehension processes for online and offline reading are similar (Coiro and Dobler, 2007). Rasmusson and Eklund (2013) pointed out that traditional literacy is one of the important online reading skills and abilities. Basic reading skills help students to recognize important concepts in hypertexts and direct them to navigate sections relevant to their goals (Salmerón and García, 2011). Offline reading comprehension abilities form an important foundation for online reading comprehension and affect how students perform when they engage in online reading.

### Conclusion

This study used eye-tracking technology and RTA techniques to examine online reading cognitive processes and comprehension performance among fifth-graders. The results show that when these students engaged in reading with goals, they adjusted their reading speed and the focus of their attention. In addition, their offline reading ability influenced their online reading performance. However, students at this learning stage, irrespective of their reading ability, found it difficult to navigate the non-linear structure of hypertexts when searching for and integrating information.

Offline reading skills and strategies still have to be taught in order to enhance the online reading abilities of elementary-school students. These students also have to be shown how to applying offline reading skills when online reading whenever appropriate, even though online reading requires more new comprehension skills and strategies than offline reading (Afflerbach and Cho, 2009; Hartman et al., 2010). Students at this learning stage have abundant opportunities to use computers and the Internet, but they do not receive sufficient instruction on what strategies they can use for online reading (Chen, 2010). It is therefore imperative that online reading strategies form part of their education. The participants in the present study rarely used search tools, were unable to construct keywords, and generally did not use search strategies. Giving students ample opportunities to engage in reading that involves searching for information helps foster their metacognitive strategies and navigation skills (Wu, 2014).

This study used a closed hypertext website to simulate the digital environment for online reading, and this is not identical to online reading on the Internet. For example, a closed system does not have the expandable and unbounded features that characterize the Internet, which is an open system. In addition, in a closed hypertext system, hyperlinks (e.g., the title hyperlinks used in this study) are built according to a clear conceptual structure or logic. However, in an open system such as the Internet, readers can discover and create new links (Coiro, 2011), which makes the online reading process more complex and hence makes it necessary to apply more reading strategies. Future studies could provide a more open and authentic online reading environment in order to simulate real online reading processes.

### Conflict of interest statement

The authors declare that the research was conducted in the absence of any commercial or financial relationships that could be construed as a potential conflict of interest.

## Footnotes

^1^The ANOVA summary tables and figures are provided on our website (https://db.tt/L9m5ZWqO).
