# Supplementary material for: Examining the online reading behavior and performance of fifth-graders: evidence from eye-movement data
Source: Front Psychol. 2015 May 28;6:665. doi: 10.3389/fpsyg.2015.00665 (PMC4446912; doi:10.3389/fpsyg.2015.00665)
Supplement: Supplementary file 1 [file Table1.PDF]

Supplementary Material:

Examining the online reading behavior and performance of fifth-graders: evidence from eye-movement data

Appendix A

The online-reading problem-solving task used in this study consisted of four different themes: the formation and structure of the Earth, the composition of biological organisms, nanoscience, and constellations. Each theme contained two multiple-choice questions and an essay question, as follows:

1. The formation and structure of the Earth
   1-1 What lies at depths between 100 and 250 km below the surface of the Earth?
   (1) the continental crust; (2) the oceanic crust; (3) the lithosphere; (4) the asthenosphere
   1-2 Which of the following is *not* one of the three main submarine topographical features?
   (1) mid-ocean ridges; (2) ocean basins; (3) continental margins; (4) oceanic trenches
   1-3 Briefly explain the relationship between fossils and sedimentary rocks. Also list at least two factors that may affect the accuracy of fossil dating.

2. The composition of biological organisms
   2-1 When Hooke first observed a thin slice of cork, he mainly saw
   (1) the cell membrane; (2) cell protoplasm; (3) the cell wall; (4) the cell nucleus
   2-2 Which part of the cell contains DNA (deoxyribonucleic acid), a molecule that encodes the genetic instructions and is the basic unit of life?
   (1) the cell membrane; (2) the cell protoplasm; (3) the cell wall; (4) the cell nucleus
   2-3 Explain the relationship between diffusion and osmosis? Give two examples of diffusion in daily life.

3. Nanoscience
   3-1 Nanotechnology is the specialized methods to manufacture materials, matters, or surfaces on the scale of
   (1) 1–1,000 nanometers; (2) 10–1,000 nanometers; (3) 1–100 nanometers; (4) 100–10,000 nanometers
   3-2 The physical properties of materials maybe change when they are reduced to the nanoscale. Which one of the following physical properties of materials *does not* change at nanoscale dimensions?
   (1) mass; (2) heat conductivity; (3) degree of curvature; (4) color
   3-3 Give two examples of nanotechnology in nature and explain how they work. Also give two examples of everyday appliances that make use of nanotechnology and explain how they work.

4. Constellations
   4-1 Which of the following celestial bodies is self-luminous?
   (1) a comet; (2) a satellite; (3) a star; (4) a planet
   4-2 What are the main components of Jovian planets?
   (1) metal and gas; (2) ice and rock; (3) gas and ice; (4) metal and rock
   4-3 How do you find stars or constellations in the sky? What factors should you pay attention to before and while you are observing stars?

Appendix B

The frequencies of using online reading strategy were calculated using an online-reading-strategy coding table (see below).

Classification	Code	Definition	Behaviors
Searching (according to eye-tracking data)	Srk_1	Entering a keyword	Type a keyword into the search box.
	Srk_2	Uses two or more keywords	Type two or more keywords into the search box.
	Srk_3	Enter an internalized keyword	In the search box, enter terms (conceptual words or keywords) that, while related to its content, are not actually found in the text.
	Src_1	Copy the text	Copying words found in the text and pasting them into the search box.
Free browsing (according to eye-tracking data)	Fb_1	Randomly browsed the text or search results	In the first viewing of an article or search result, randomly looked around and didn't finish reading sentences/paragraphs before browsing other content.
	Fb_2	Looks at either the text or search results only from top to bottom (and not from side to side)	Looks at either the text or search results from top to bottom (and not from side to side).
Comprehension monitoring (according to RTA data)	M_1	Assessing whether oneself understands the article	Judge from the subject's responses whether or not they understood the text.
	M_2	Figuring out the parts of an article one doesn't understand, such as by reading it again, slowing down one's reading speed, or finding/using other strategies	Judge from the subject's responses whether or not the subject sought to elucidate confusing content.
	M_3	Asking a question of the article	Judge from the subject's responses whether or not they asked questions of the article.
	M_4	Identify confusing content and clarify doubts	Judge from the subject's responses whether or not they identified confusing content and clarified doubts.
